# *In silico* deconvolution and purification of cancer epigenomes

**DOI:** 10.18632/oncoscience.346

**Published:** 2017-04-14

**Authors:** Martí Duran-Ferrer, Renée Beekman, José I. Martín-Subero

**Affiliations:** Biomedical Epigenomics Group, Institut d'Investigacions Biomèdiques August Pi i Sunyer (IDIBAPS), Universitat de Barcelona, Barcelona, Spain

**Keywords:** deconvolution, epigenetic profiling, DNA methylation, cancer, tumor purity

**Figure 1:**
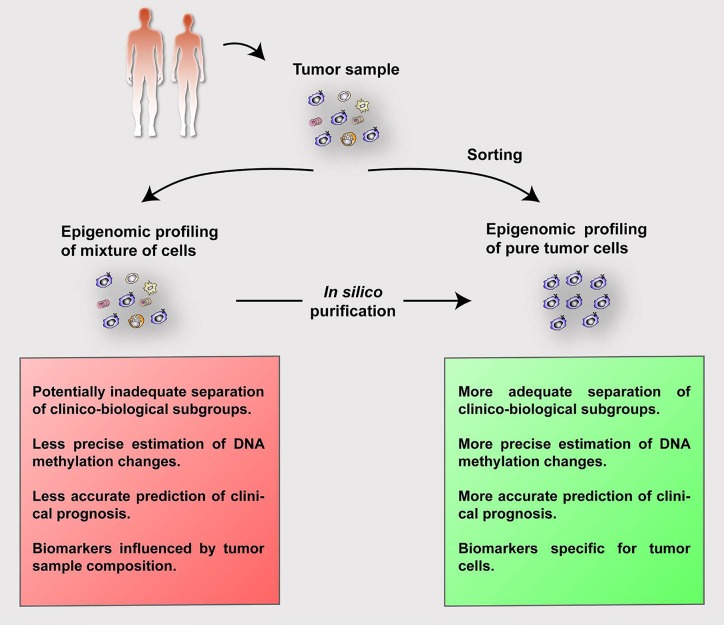
Summary of the process and advantages of analyzing purified (either by cell sorting or *in silico*) epigenomic signatures of tumor cells

Large scale epigenomic initiatives are generating vast amounts of epigenomic profiles from normal and neoplastic cells, including whole genome maps of DNA methylation, histone modifications, chromatin accessibility and the 3D chromatin structure [1]. The effect of tumor cell content is well taken into consideration in fields like genomics, but its impact on epigenomic data remains poorly understood. Tumor samples frequently contain an admixture of neoplastic cells and a nonnegligible proportion of healthy cell subtypes from the tumor microenvironment (Figure 1, top), which themselves also show distinct epigenomic profiles. Thus, epigenomic analyses using samples with varying tumor purity in combination with varying proportions of microenvironmental cells can eventually lead to inaccurate biological and clinical interpretations.

We recently performed a detailed analysis of the DNA methylome of mantle cell lymphoma (MCL), a clinically heterogeneous B-cell tumor [2]. Our initial experimental design included samples with a high tumor cell content (median 89%, range 56-100%). However, two referees of the submitted manuscript questioned whether our results and interpretation were influenced by tumor cell content. This criticism triggered a series of analyses and indeed, we observed that tumor cell content greatly affected the DNA methylation estimates and all subsequent downstream biological and clinical associations. In MCL, the major sites of presentation are lymph node (LN) and peripheral blood (PB). Tumor cells from PB can be purified by cell sorting (Figure 1, right). However, if the lymphoma presents in solid tissues, samples are routinely processed in pathology departments as formalin- fixed paraffin-embedded blocks or cryoblocks, which hampers any possibility of cell sorting (Figure 1, left). To remove the effect of contamination of non-tumoral cells from the MCL samples, we adapted the statistical framework proposed by Houseman and coworkers [3] to estimate the proportions of cell subpopulations based on DNA methylation patterns. Next, we used the *in silico* estimated percentages of tumor and microenvironmental cells of each MCL sample, and removed the contribution of the microenvironment to end up with purified DNA methylation values from tumor cells. We repeated all the downstream analyses with corrected DNA methylation values, and all our biological and clinical interpretations became more clear than with uncorrected values (Figure 1, bottom). These included a better separation of two clinico-biological MCL subgroups with different cellular origin and a better estimation of the epigenetic drift (i.e. number of changes per case as compared to hematopoietic precursor cells), which in turn is highly associated with clinical outcome.

In order to correct the DNA methylation values *in silico*, few requirements should be met. First, to predict the purity of tumor samples, a DNA methylation signature of tumor cells related to its cellular origin should be available. This allows to distinguish tumor cells from the microenvironment. In our case, all MCLs contained a clear signature of their B-cell origin. Of note, in some instances, like in multiple myeloma, tumor cells tend to erase the B-cell signature [4] which would make the *in silico* deconvolution and purification inaccurate. Second, the proportion of tumor cells should be much higher than that of their normal cell counterparts. In MCL the proportion of normal B cells is negligible as compared to neoplastic B cells, and therefore the total estimated B-cell fraction could be taken as a surrogate for the tumor cell fraction. Third, we observed that the tumor cell fraction should be sufficiently high, i.e. more than 50%, to accurately correct DNA methylation values. Fourth, reference DNA methylation patterns of purified normal cell types from the tumor microenvironment should be available to allow for proper estimations of their respective fractions and to accurately remove their contribution to the methylation signal of the unpurified sample. In this context, it is worth mentioning that cellular composition can also be estimated using reference-free methods, which have the virtue of allowing deconvolution of methylation signals into its constituents in complex tissues with unknown cell types [5]. However, reference-based methods are superior to reference-free ones if the cellular composition of the samples is known and DNA methylomes for the main cell types are available [6]. In our study, PB samples could be accurately deconvoluted due to the availability of DNA methylation profiles of the major cell subtypes in PB. However, in the case of LN samples, some cell subpopulations were not available, such as macrophages and endothelial cells, and we recognize that deconvolution was less accurate for those samples.

In this editorial, we aimed at highlighting the importance of tumor purity for DNA methylation studies. Ideally, tumor cells should be sorted in the laboratory or, if not possible, adequate bioinformatic pipelines should be applied to deconvolute and purify tumor cell DNA methylation estimates *in silico*. The benefit of the proposed analytic strategy is multiple. On the one hand, it allows to estimate the composition of the tumor microenvironment, which has been shown to be biologically and clinically important in many tumor entities [7]. On the other hand, this analysis allows for a more accurate characterization of DNA methylation profiles of tumors cells and leads to more robust biological interpretations and clinical associations. Finally, it paves the way for the identification of diagnostic and/or prognostic biomarkers that are not influenced by tumor sample composition.
